# Novel Susceptibility Locus at 22q11 for Diabetic Nephropathy in Type 1 Diabetes

**DOI:** 10.1371/journal.pone.0024053

**Published:** 2011-09-01

**Authors:** Maija Wessman, Carol Forsblom, Mari A. Kaunisto, Jenny Söderlund, Jorma Ilonen, Riitta Sallinen, Tero Hiekkalinna, Maija Parkkonen, Alexander P. Maxwell, Lise Tarnow, Hans-Henrik Parving, Samy Hadjadj, Michel Marre, Leena Peltonen, Per-Henrik Groop, on behalf of the FinnDiane Study Group

**Affiliations:** 1 Folkhälsan Research Center, Folkhälsan Institute of Genetics, Biomedicum Helsinki, Helsinki, Finland; 2 Biomedicum Helsinki, Research Program in Molecular Medicine and Institute for Molecular Medicine Finland FIMM, University of Helsinki, Helsinki, Finland; 3 Division of Nephrology, Department of Medicine, Helsinki University Central Hospital, Helsinki, Finland; 4 Department of Clinical Microbiology, University of Eastern Finland, Kuopio, Finland; 5 Immunogenetics Laboratory, University of Turku, Turku, Finland; 6 Unit of Public Health Genomics, National Institute for Health and Welfare, Helsinki, Finland; 7 Nephrology Research Group, Centre for Public Health, Queen's University of Belfast, Belfast, Northern Ireland, United Kingdom; 8 Steno Diabetes Center, Gentofte, Denmark; 9 Department of Medical Endocrinology, Rigshospitalet, University of Copenhagen, Copenhagen, Denmark; 10 Faculty of Health Science, Aarhus University, Aarhus, Denmark; 11 Service de Médecine Interne, Endocrinologie et Maladies Métaboliques, Centre d'Investigation Clinique, Inserm CIC0802, CHU de Poitiers, Poitiers, France and Université de Poitiers, Poitiers, France; 12 Department of Endocrinology, Diabetology and Nutrition, Bichat-Claude Bernard University Hospital, Paris, France; 13 Department of Human Genetics, Wellcome Trust Sanger Institute, Wellcome Trust Genome Campus, Hingston, United Kingdom; South Texas Veterans Health Care System, United States of America

## Abstract

**Background:**

Diabetic nephropathy (DN) affects about 30% of patients with type 1 diabetes (T1D) and contributes to serious morbidity and mortality. So far only the 3q21–q25 region has repeatedly been indicated as a susceptibility region for DN. The aim of this study was to search for new DN susceptibility loci in Finnish, Danish and French T1D families.

**Methods and Results:**

We performed a genome-wide linkage study using 384 microsatellite markers. A total of 175 T1D families were studied, of which 94 originated from Finland, 46 from Denmark and 35 from France. The whole sample set consisted of 556 individuals including 42 sib-pairs concordant and 84 sib-pairs discordant for DN. Two-point and multi-point non-parametric linkage analyses were performed using the Analyze package and the MERLIN software. A novel DN locus on 22q11 was identified in the joint analysis of the Finnish, Danish and French families by genome-wide multipoint non-parametric linkage analysis using the Kong and Cox linear model (NPL_pairs_ LOD score 3.58). Nominal or suggestive evidence of linkage to this locus was also detected when the three populations were analyzed separately. Suggestive evidence of linkage was found to six additional loci in the Finnish and French sample sets.

**Conclusions:**

This study identified a novel DN locus at chromosome 22q11 with significant evidence of linkage to DN. Our results suggest that this locus may be of importance in European populations. In addition, this study supports previously indicated DN loci on 3q21–q25 and 19q13.

## Introduction

Diabetic nephropathy (DN) is a major complication in patients with type 1 diabetes (T1D) and contributes to serious morbidity and mortality. It affects about one-third of the patients [1], [2] and is characterized by high blood pressure, proteinuria, a progressive decline in renal function and an increased risk of cardiovascular disease.

The incidence of DN reaches its peak after 20 years of diabetes [1], [2]. The observed incidence pattern and familial clustering [3]–[6] suggest an important role for genetic factors in the development and progression of DN. Although the majority of sib-pairs with T1D are discordant for DN, its presence in one sibling doubles the risk (λ = 2.1) for the other sibling with diabetes [5], [6].

The chromosome region 3q21–q25 has been linked to DN in patients with T1D in several genetic studies [7]–[10]. Apart from a genetic variant recently identified at 3q22 [11] no other genetic variants that convincingly predispose to DN in patients with T1D have been identified [12], [13]. Findings from numerous case-control candidate gene association studies have to a large extent been contradictory and the studies have often been performed in a small number of subjects and with a relatively limited set of markers analyzed. Results from a recent genome-wide association study (GWAS) [14] suggest novel pathways in the pathogenesis of DN. In general, a common trend in GWAS data has been that the best signals are detected in regions not previously studied using the candidate gene approach. This suggests that different study designs and approaches are needed to detect susceptibility loci for complex diseases such as DN.

The aim of this study was to search for susceptibility loci for DN in Finnish, Danish and French T1D families using a genome-wide linkage strategy. We found a new susceptibility locus and were able to replicate two previously identified loci.

## Methods

### Ethics Statement

All participants provided written, informed consent. Approvals to conduct the research were obtained from the Helsinki University Central Hospital Ethics Committee, Finland, the Ethical Committee of Copenhagen, Denmark, and the Angers University Ethics Committee, France. The study was in accordance with the principle of the Helsinki Declaration.

### Families

Altogether 175 Finnish, Danish and French families with T1D were studied. The families were mainly nuclear families including one or both parents and siblings concordant or discordant for DN or solely sib-pairs concordant or discordant for DN. In addition, sib-pairs with T1D and normal renal status were included. Patients with DN were identified among those with age of onset of T1D at 35 years or less and who had had T1D for at least 10 years. The distribution of siblings per family and the number of parents and other relatives studied are presented in Table 1.

**Table 1:** Characteristics of the families genotyped for the linkage study.

	Finnish	Danish	French	Total
**N of genotyped individuals**	351	114	91	556
Siblings	250	97	74	421
DN+	63	61	34	158
DN−	130	35	38	203
Parents	87	17	16	120
DN+	1	-	1	2
DN−	-	-	1	1
other relatives	14	-	1	15
DN+	2	-	1	3
DN−	7	-	-	7
**N of families**	94	46	35	175
2 parents genotyped	31	8	7	46
1 parent genotyped	25	1	2	28
0 parents genotyped	38	37	26	101
1 sibling with T1D	1	0	0	1
2 siblings with T1D	82	41	32	155
3–4 siblings with T1D	11	5	3	19
genotyped individuals/family, mean (range)	3.7 (2–11)	2.5 (2–5)	2.6(2–5)	
genotyped siblings/family, mean (range)	2.7 (2–5)	2.1(2–3)	2.1(2–3)	
genotyped siblings with T1D/family, mean (range)	2.1 (1–4)	2.1(2–3)	2.1(2–3)	

Sib-pairs	Finnish	Danish	French	Total
DN+/+	18	15	9	42
DN−/+	32	36	16	84
DN−/−	56	3	14	73
T1D+/+ pairs	118	56	41	215

N = number; DN = diabetic nephropathy; T1D = Type 1 Diabetes.

The Finnish families were recruited by the Finnish Diabetic Nephropathy (FinnDiane) Study Group [15]. All adult T1D patients, treated at the FinnDiane centers around the country, were invited to participate in the study. The patients were asked about their family history and a special effort was made to recruit families with more than one patient with T1D. If the parents were not alive, non-diabetic siblings were invited to participate in the study. A total of 94 Finnish families were recruited 93 of which had at least two siblings with T1D (Table 1).

In Denmark, a nationwide survey of the Danish Registry of Diagnosis was used to identify patients diagnosed with T1D and DN. After verification of patient records, a letter of invitation was sent to all patients with a diabetic sibling. A total of 46 families with at least two siblings with T1D were included in the study (Table 1).

The French and Belgian families (from now on indicated as French families) were collected on the occasion of the Genesis (Genetic Nephropathy Sib pair Study) France-Belgium study [16]. Family information was obtained from medical examination and/or structured questionnaire. A total of 35 families with at least two siblings with T1D were included in the study (Table 1).

### Diagnoses

The diagnosis of T1D was based on age at onset less than 35 years and permanent insulin treatment initiated within one year of the diagnosis. Normal renal status, i.e. normoalbuminuria (DN−), was defined as an albumin excretion rate (AER) <20 µg/min or <30 mg/24 h in two out of three consecutive overnight or 24-hour urine collections. Incipient nephropathy (microalbuminuria) was defined as an AER 20–200 µg/min or 30–300 mg/24 h and overt nephropathy (proteinuria, DN+) as an AER >200 µg/min or >300 mg/24 h. The patients on dialysis or with a kidney transplant due to diabetic nephropathy were also considered to have nephropathy (DN+). Retinopathy was defined as the presence or absence of retinal laser treatment. Cardiovascular hard end-points were defined as a history of myocardial infarction, coronary revascularization procedure, stroke or amputation. The clinical characteristics of the subjects with T1D in each sample are presented in Table 2.

**Table 2:** Clinical characteristics of examined diabetic siblings according to renal status.

	Finnish		Danish		French	
	Diabetic siblings		Diabetic siblings		Diabetic siblings	
Characteristics	Normo-albuminuria	Macroalbuminuria and ESRD	Normo-albuminuria	Macroalbuminuria and ESRD	Normo-albuminuria	Macroalbuminuria and ESRD
N (M/F)	130 (70/60)	63 (38/25)	35 (20/15)	61 (34/27)	38 (20/18)	34 (18/16)
Age at examination (yrs)	40.7±12.7	42.3±9.4	43.6±10.6	43.4±9.1	45.9±10.2	41.7±15.6
Age at onset of diabetes (yrs)	16.3±11.0	11.8±7.9	18.9±10.5	14.0±8.1	18.8±8.8	14.7±10.7
Duration of diabetes (yrs)	24.4±11.8	30.6±8.5	24.7±10.1	29.4±9.5	27.1±8.4	27.0±13.9
Body mass index (kg/m^2^)	25.3±3.6	26.9±5.0	23.3±3.3	23.8±3.2	NA	NA
HbA_1c_ (%)	8.3±1.6	8.8±1.7	8.9±1.9	9.5±1.5	NA	NA
Retinopathy (%)	16.3	90.2	27.0	66.7	22.9	77.0
CVD Hard (%)	3.8	33.9	NA	NA	NA	NA
Systolic blood pressure (mmHg)	133±19	144±19	137±19	152±20	NA	NA
Diastolic blood pressure (mmHg)	78±10	82±9	77±7	80±11	NA	NA

N = Number; M = Male; F = Female; ESRD = End stage renal disease; NA = Not Applicable.

Data are summarized as mean ± standard deviation.

### Genotyping of microsatellite markers

Genotyping was performed at the Finnish Genome Center. The procedure was conducted using the MegaBACE (GE Healthcare Life Sciences, Piscataway, NJ, USA) or the ABI genotyping systems (Applied Biosystems, Foster City, CA, USA) and the LMS-MD10 microsatellite marker set (Applied Biosystems) as previously described [17]. The marker set consisted of 384 markers with a 9.5 cM average inter-marker distance and covered all autosomes and the X chromosome. A further 13 markers were genotyped at chromosome 3q in the Finnish sample resulting in a coverage of 2.4 cM average intermarker distance from marker D3S1558 to D3S3668. All three sample sets were further genotyped with four markers at 22q resulting in a coverage of 6.9 cM average inter-marker distance from marker D22S420 to D22S1163. The Mendelian inconsistencies of the genotypes were detected using the PedCheck1.1 program [18].

### Genotyping of the HLA loci

HLA DQA1-DQB1 haplotypes and DRB1*04 subtypes of DQB1*0302 positive haplotypes were screened in the Finnish families using the PCR-based lanthanide-labelled oligonucleotide hybridization and time-resolved fluorometry detection as described earlier [19].

### Statistical analyses

Initially, two-point non-parametric affected sib-pair (ASP) (DN+/DN+) linkage analysis was performed genome-wide. This analysis, as well as the transmission-disequilibrium-test (TDT), were performed using the ANALYZE program package implemented in the AUTOGSCAN software tool [20]. The identity-by-descent (IBD) of each sib-pair was estimated using the Merlin software program [21]. Multipoint non-parametric linkage [22] analyses were also performed using the Merlin program [21]. NPL_pairs_ was used to analyze affected pairs (DN+/DN+). In order to use the information from unaffected individuals as well, a NPL quantitative trait linkage statistic (NPL_qtl_) was applied as described previously [17], [23]. Affected individuals (DN+) were coded as “1”, unaffected individuals (DN−) as “0” and those with missing phenotypes as “x” (affection status unknown, patients with microalbuminuria and non-diabetic individuals) in this analysis. MERLIN program uses Z-score statistics to construct a likelihood ratio test for linkage and define a LOD score statistic using the procedure as previously described [24]. The Kong and Cox linear model was applied to evaluate the evidence for linkage. This model is designed to identify small increases in allele sharing spread across a large number of families as is usually expected in a complex disease. In all analyses, allele frequencies were estimated by counting among all individuals. The AUTOSCAN 1.0.3. software tool was used to automate the genome-wide analyses [20].

### Significance limits

We estimated the significance thresholds for ASP analysis of 400 markers using the formulae presented by Feingold et al. [25]. The Lander and Kruglyak threshold for significant evidence of linkage corresponding to a standard LOD score of 3.63 (p = 0.000022) [26] is decreased to a LOD score of 3.05 (p = 0.00009). Similarly, the threshold for suggestive linkage is reduced from p = 0.00074 (LOD score of 2.19) to p = 0.0023 (LOD score of 1.74). These theoretically-derived thresholds are consistent with those obtained via simulation by others [23], [27], [28].

## Results

### Genome-wide population-specific linkage analyses in concordant sib-pairs

**Figure 1:**
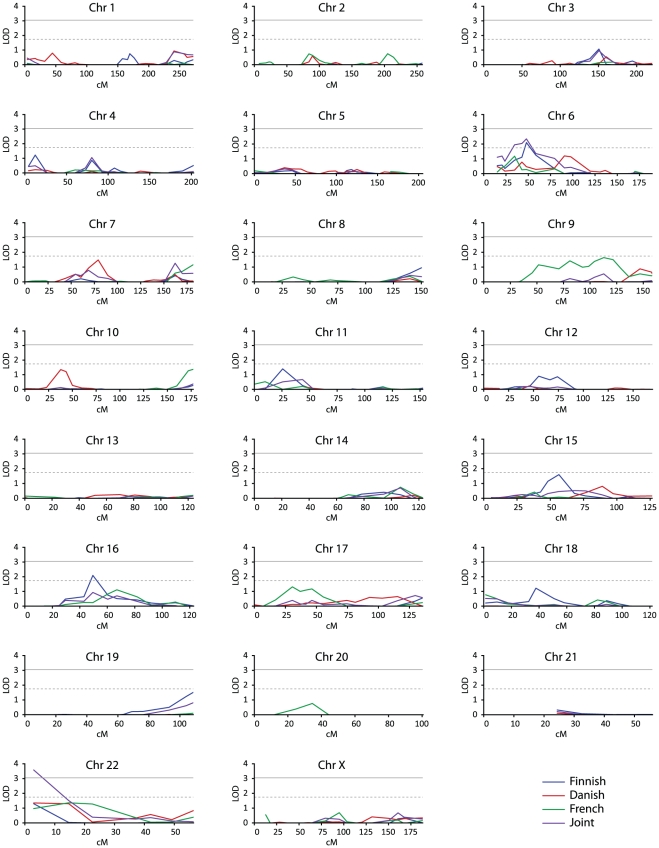
Results of the genome-wide linkage analysis for diabetic nephropathy. The graphs show values for the Finnish (blue curves), Danish (red curves), French (green curves) and the joint study samples (purple curves) in the multipoint NPL_pairs_ analysis. For each chromosome, genetic distance (cM) is plotted on the x-axis against the LOD score on the y-axis. Lower horizontal dotted lines represent suggestive evidence (LOD≥1.74) and upper horizontal lines show threshold for significant evidence (LOD≥3.05) of linkage by Feingold *et al.* [25] criteria.

We first wanted to identify regions linked to DN in each population sample separately utilizing the concordant DN+/DN+ sib-pairs. Summary of the best ASP and NPL results is presented in Table 3. Plotted results of the genome-wide NPL_pairs_ are shown in Figure 1.

**Table 3:** Summary of the non-parametric linkage results for diabetic nephropathy on the regions showing significant or suggestive evidence of linkage.

				Non-parametric two-point		Non-parametric multipoint	
	Chr	cM	Marker^a^	ASP LOD	All-ASP LOD	NPL_pair_ LOD	NPL_qtl_ LOD
**Finnish**	5p15	36.73	D5S416	0.13	0.95	0.18	**1.94**
	5p14	46.2	D5S419	0.07	1.20	0.20	**2.06**
	6p21	47.9	D6S276	**2.31**	0.43	**2.10**	0.24
	15q15.1	42.62	D15S994	0.08	1.28	0.42	**1.74**
	16p12	49.5	D16S3068	**1.82**	0.28	**2.09**	0.28
	17q25.3	129.62	D17S784	0.22	**2.04**	0.34	1.35
	22q11	2.96	D22S420	**2.09**	0.07	1.30	0.32
**Danish**	22q11	2.96	D22S420	0.66	0.59	1.35	1.15
**French**	6p23	34.61	D6S289	1.36	**2.61**	1.18	1.20
	9p21.2	51.5	D9S161	0.83	**1.94**	1.15	**2.19**
	22q11	2.96	D22S420	0.33	0.00	0.97	0.01
**Joint**	6p23–p22.3	34.61	D6S289	1.79	**2.41**	1.95	1.49
	6p22.3	42.83	D6S422	1.06	0.27	**2.04**	0.27
	6p21	47.93	D6S276	**2.82**	0.37	**2.35**	0.04
	7q21.11	162.09	D7S669	0.14	**1.92**	0.23	0.75
	22q11	2.96	D22S420	**2.78**	0.31	**3.58**	0.86
**Joint including finemapping markers**	22q11	2.96	D22S420	**2.78**	0.31	**2.19**	0.60
	22q11.21	5.8	D22S427^a^	0.51	0.12	1.69	0.82
	22q11.21–q22	14.68	D22S539	0.47	0.82	1.43	1.49
	22q11.23	18.8	D22S1174^a^	1.49	1.13	1.28	1.20
	22q11.23–q12.1	22.59	D22S315	0.07	0.6	0.86	1.37
	22q12.1	24.38	D22S1154^a^	0.15	0.64	1.09	1.33
	22q12.1	30.54	D22S1163^a^	0.71	0.74	0.84	0.52

Chr = chromosome; cM = centiMorgan; ASP = two-point affected sib-pair LOD score; NPL = multi-point non-parametric LOD score based on Merlin sib-pair analysis; NPL_qt l_ = multi-point non-parametric LOD score based on Merlin qtl-analysis;

a additional markers genotyped on chromosome 22q.

Results on D22S420 are shown in each population and results after mapping four additional markers on chromosome 22q11–q12 in the joint sample set.

In the Finnish study sample no chromosomal region showed significant evidence of linkage to DN in the ASP analysis. Suggestive evidence of linkage was, however, found between DN and four chromosomal regions: 6p21 (ASP LOD score 2.31), 16p12 (ASP LOD score 1.82), 17q25.3 (all-ASP LOD score 2.04), and 22q11 (ASP LOD score 2.09) (Table 3). Nominal evidence of linkage (only LOD Scores above 1.00 are considered) was found to eight additional chromosomal regions (Table 3, Table S1). Two of these regions, i.e. 3q24 and 19q13, are of interest since they have been indicated as DN loci in previous studies [7]–[10].

In order to pool information from multiple markers we also performed a multipoint, nonparametric NPL_pairs_ analysis. The test indicated suggestive evidence of linkage between DN and 6p21 (NPL_pairs_ LOD score 2.10), and 16p12 (NPL_pair_ LOD score 2.09).

In the Danish sample, no chromosomal region showed significant or suggestive evidence of linkage to DN in the ASP analysis. Nominal evidence of linkage was found to six chromosomal regions (Table S1). Multipoint, nonparametric NPL_pairs_ analysis indicated several loci also supporting linkage to 22q11.

In the French sample, ASP analyses showed suggestive evidence of linkage between DN and 6p23 (all-ASP LOD score 2.61) and 9p21 (all-ASP LOD score 1.94). Nominal evidence of linkage was found to five additional chromosomal regions including 22q (Table S1). Results of the multipoint, nonparametric NPL_pairs_ analysis supported evidence of linkage to most of these regions and also indicated linkage to 7q36 and 16q12 (Table S1).

### Genome-wide joint analysis of the concordant Finnish, Danish, and French sib-pairs

Results of a joint ASP analysis of the Finnish, Danish and French families showed suggestive evidence of linkage between DN and 22q11 (ASP LOD score 2.78, Table 3). When multipoint NPL_pairs_ analysis was applied, the linkage result at 22q11 improved showing significant evidence of linkage between D22S420 and DN (NPL_pairs_ LOD score 3.58, Table 3). Joint ASP analysis also indicated suggestive evidence of linkage between DN and 6p21 (ASP LOD score 2.82). The results from the joint analyses are presented in Figure 1, Table 3 and Table S1.

### HLA-locus at 6p21 in the Finnish sample

We decided to study the 6p21 region further and genotyped the Finnish patients for the common HLA DR-DQ haplotypes. We also analyzed the Finnish sample set for T1D, and found significant evidence of linkage to the HLA-region on 6p21 (NPL_pairs_ LOD score of 5.44) providing validation that our patient population is comprised of T1D patients [29]. TDT analysis of the HLA locus gave an overall p-value of 9.8·10^−7^ for DN. However, this was mainly driven by the major known T1D risk haplotype DRB1*0401_DQB1*0302, p-value for this allele was 2.6·10^−5^ for DN. All DN+/DN+ pairs are by definition also concordant for T1D.

### Identical-by-descent (IBD) estimation

IBD analysis estimates the probabilities that pairs of individuals share marker alleles identical by descent. Probabilities that a given sib-pair will share 0, 1 or 2 alleles IBD are 25%, 50% and 25%. The estimation of the IBD was performed for the loci showing either significant (22q11) or suggestive (6p22, 9p21, 16p12) evidence of linkage in the ASP analysis and for the two loci previously indicated in DN (3q21–q25 and 19q13). IBD estimation indicated true linkage between DN and 22q11, 3q24, 9p21, 16p12 and 19q13 (Table 4).

**Table 4:** Identity- by-descent (IBD) sharing and results on two-point Affected Sib Pair (ASP) linkage analysis for the most interesting diabetic nephropathy susceptibility loci.

			DN+/+			DN+/−				
Chr	Marker	Population	z0	z1	z2	z0	z1	z2	ASP LOD	All-ASP LOD
22	D22S420	Finland	0.12	0.43	0.45	0.22	0.51	0.27	2.09	0.06
		Denmark	0.10	0.52	0.38	0.22	0.67	0.11	0.66	0.59
		France	0.04	0.70	0.25	0.16	0.58	0.26	0.33	0.00
		All	0.10	0.52	0.38	0.21	0.59	0.20	2.78	0.31
22	D22S539	Finland	0.24	0.53	0.24	0.31	0.51	0.18	0.00	0.46
		Denmark	0.07	0.54	0.39	0.28	0.51	0.20	0.72	0.19
		France	0.04	0.43	0.53	0.11	0.48	0.41	1.59	0.15
		All	0.13	0.51	0.36	0.26	0.51	0.23	0.47	0.82
3	D3S1569	Finland	0.08	0.48	0.44	0.22	0.65	0.12	0.76	1.08
6	D6S276	Finland	0.09	0.30	0.61	0.09	0.36	0.55	2.31	0.43
9	D9S161	France	0.02	0.49	0.49	0.20	0.57	0.23	0.83	1.94
16	D16S3068	Finland	0.06	0.47	0.47	0.24	0.40	0.35	1.82	0.28
19	D19S210	Finland	0.07	0.43	0.50	0.16	0.59	0.26	1.61	0.11

DN+/+ = concordant for diabetic nephropathy; DN+/− = discordant for diabetic nephropathy; Chr = chromosome; z0 = 0 alleles; z1 = 1 allele; z2 = 2 alleles.

Figures represent probabilities of sharing either 0, 1 or 2 alleles presented as mean values.

### NPL_qtl_ analysis on concordant and discordant sib-pairs

In order to obtain additional linkage information from unaffected individuals (DN− i.e. normoalbuminuric T1D patients), we used a non-parametric quantitative trait linkage NPL_qtl_ Z-score statistic. In the Finnish sample suggestive evidence of linkage between DN and loci on 5p15-p14 and 15q15 was detected (Table 3). Two consecutive markers at 5p15-p14 showed linkage (NPL_qtl_ LOD scores 2.06 at 46 cM and 1.94 at 37 cM). The linkage peak at 15q15 (NPL_qtl_ LOD score 1.74 at 43 cM) was surrounded by two markers also indicating linkage (LOD scores 1.42 and 1.56, Table S1). Nominal evidence of linkage was found to four other chromosomal regions including 22q11 (Table S1).

In the Danish sample nominal evidence of linkage was detected to 5q33.2 and 22q11 (Table S1).

In the French sample, suggestive evidence of linkage between 9p21 and DN (NPL_qtl_ LOD score 2.19) was detected (Table 3). Nominal linkage was found to two markers surrounding the 9p21 peak and to a locus on 6p23 (Table S1).

When all three sample sets were analyzed jointly nominal evidence of linkage to five different chromosomal regions, including 22q11, was detected (Table 3 and Table S1).

### Fine-mapping the loci on 22q11 and 3q24

**Figure 2:**
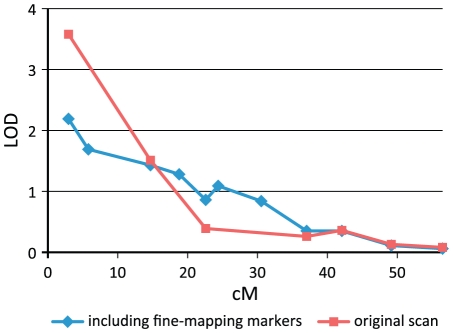
Results of multipoint linkage analysis on chromosome 22q. The graphs show the NPL_pairs_ results on diabetic nephropathy obtained in the joint sample set in the original genome-wide screen (red curve) and after fine-mapping markers were included in the analysis (blue curve).

Since the joint analysis of the Finnish, Danish and French families showed significant evidence of linkage to 22q11, we genotyped four additional markers on 22q11–q12 in all three study samples to increase the linkage information across the implicated region. The highest linkage peak was found at 3 cM (D22S420, NPL_pairs_ LOD score of 2.19) even after these markers were included in the analysis. The results are detailed in Figure 2 and Table 3.

Since the 3q21–q25 region has been suggested as a DN susceptibility region in several previous studies [7]–[10], we decided to fine-map the region in the Finnish sample by genotyping 13 additional markers (data not shown) around our linkage peak at 3q24 (150.6 cM, NPL_qtl_ LOD score 1.36, D3S1569). When the 13 markers were included in the analysis, the highest peak was found at 152.3 cM (NPL_qtl_ LOD score of 1.05, D3S1593).

## Discussion

The present study was conducted in three different population samples, the Finnish, the Danish, and the French, among which T1D is most common in the Finns [30]. We detected significant evidence of linkage (NPL_pairs_ LOD score 3.58) between DN and 22q11 in the joint analysis of the Finnish, Danish and French T1D families. When analyzed separately, the samples showed either suggestive or nominal evidence of linkage between DN and 22q11. The IBD pattern in all three samples indicated that concordant sib-pairs share two alleles more often than expected while discordant pairs do not share alleles more often than expected. For example, when viewing marker D22S420 in the Finnish sample 45% of the DN+/DN+ pairs share two alleles while 27% of the DN+/DN− pairs share two alleles. This difference indicates that our finding is most likely due to DN and not to T1D. Similar effect was observed in the joint sample where the corresponding values were 38% and 20%. After fine-mapping the region around the 22q11 locus the best linkage signal was detected at 3 cM (D22S420) and this locus does not overlap with any previously reported DN locus detected in patients with T1D. On the other hand, a locus approximately 20 Mb apart from our susceptibility region has in the FIND Study recently been linked to urinary albumin∶creatinine ratio in Mexican-Americans with diabetes [31]. This region contains the *MYH9* (myosin, heavy polypeptide 9, non-muscle) and *APOL1* (Apolipoprotein L1) genes associated in African-Americans with diabetic or nondiabetic nephropathy [32]–[34].

The 22q11 region contains over 60 protein coding genes. Two of these are especially relevant candidates for DN: *IL-17RA* encodes interleukin 17 receptor precursor protein, a ubiquitous type I membrane glycoprotein that binds with low affinity to interleukin 17A. Interleukin 17A and its receptor play a pathogenic role in many inflammatory and autoimmune diseases such as rheumatoid arthritis [35]. Inflammation is a major pathogenic mechanism in DN, including such proinflammatory molecules as cytokines IL-1, IL-6, and IL-8 [36], [37]. *SLC25A18* (solute carrier family 25, member 18) nuclear gene encoding mitochondrial protein (GC2) is involved in the transport of glutamate across the inner mitochondrial membrane [38]. It is expressed in various tissues including brain, pancreas, liver and kidney. Previous studies have revealed that polymorphisms in other solute carriers might contribute to genetic susceptibility to DN in type 2 diabetes in Asian populations (T2D) [39], [40]. The 22q11 linkage region also contains some segments coding for microRNAs (miRNAs, small noncoding RNA molecules) which regulate gene expression. Recent evidence indicates that miRNAs in general may be involved in the pathogenesis of diabetes [41].

The 22q11–q12 region also contains low-copy repeats and is known for its susceptibility to genomic rearrangements due to non-allelic homologous recombination. Various syndromes including the velo-cardia-facial/diGeorge syndrome, the cat-eye syndrome, schizophrenia, and glial tumors of the brain [42]–[44] have been linked to this region. Copy number variants as such account for a major proportion of human genetic polymorphism but their role in genetic susceptibility to common diseases such as diabetes is still unknown.

In addition to the 22q11 locus, our study further confirms the previously reported susceptibility loci for DN in T1D on 3q21–q25 and 19q13. Including our report, 3q21–3q25 has now been indicated as a susceptibility region for DN in four different populations: the American Caucasians [7], [8], Finns [9], [11], Danish (this study) and Russians [10]. Existing data implies that the 3q21–q25 region may contain several loci involved in DN. Future studies will define the role of these loci in DN across populations. The locus found on 19q13 overlaps with a region recently reported in a study on 100 American Caucasian sib-pairs discordant for DN [8]. The SNP rs306450 (at 61.2 Mb) indicating linkage in the study by Rogus et al. is 400 kb apart from the marker D19S210 showing linkage to DN in the Finnish sample. The area contains several genes coding for zinc finger proteins that are relevant candidates for further studies [45].

In addition to these three susceptibility loci, we identified in each population other possible DN loci. In the French sample two susceptibility regions were found on chromosome 9. The susceptibility region on 9p21.3 has in previous studies shown to harbor two major disease susceptibility loci: one for T2D [46], [47] and one for coronary artery disease and myocardial infarction [48], [49]. On the 9q21–q31 susceptibility region several markers indicated linkage to DN. At least two previous studies have indicated association between diabetic nephropathy and this region. Common SNPs near the *FRMD3* (FERM domain containing 3) gene have been associated with DN in Northern American populations with T1D [14]. *AUH* gene (AU RNA binding protein/enoyl-Coenzyme A) on the other hand, has been implicated in T2DM-ESRD in African Americans [50]. In the Finnish sample susceptibility loci on 16p12 and 15q14–q22 were identified. The 16p locus is 13 Mb apart from a locus suggested to affect duration of diabetes to T2DM-ESRD in African patients [51]. On 15q several markers indicated linkage to DN. Also the Find Study [31] has reported a possible DN susceptibility locus in American Indians in this region. In the Danish sample two consecutive markers on 10p12–p13 indicated linkage to DN close to a region reported previously in the Irish T1D sample [8], [52].

The strength of our study design is the use of both concordant and discordant sib-pairs as well as other relatives. This is evident especially when we view the Finnish sample and the loci at 22q11, 3q24 and 5p14–p15. The locus at 22q11 was detected in the analysis of the concordant sib-pairs. The two other loci surfaced in the NPL_qtl_ analysis that is able to retrieve information also from unaffected individuals thus increasing the number of the families in the analysis. We also looked at the IBD sharing patterns on several loci. The results of this analysis support the loci on 22q11, 3q24, 9p21, 16p12, and 19q13 as true DN loci. Linkage to the 6p21 HLA-region, on the other hand, is probably due to T1D since 61% of the DN+/DN+ pairs and 55% of the DN+/DN− pairs share both alleles.

A limitation in our study is the sample size. The power to detect susceptibility loci with moderate effects is relatively low, especially when each sample is studied alone. However, all the samples are part of thoroughly characterized and prospectively followed cohorts that have generated a large amount of clinically useful information and contributions to our understanding of the pathogenesis of DN.

The two GWAS performed to date have detected DN susceptibility regions on chromosomes 9q [14], 11p [14] and 10p [52] in T1D. It is not surprising that 22q11 was not detected in these studies. While GWAS are effective in detecting common variants with low or medium effect in a large number of unrelated cases, linkage studies can be efficient in identifying families enriched with rare susceptibility variants. Another advantage of linkage analysis is that the likelihood of false positive findings due to population stratification is reduced. Furthermore, the possibility to test allelic transmission decreases the number of Mendelian inconsistent genotypes.

In summary, we found significant evidence of linkage between a novel locus on 22q11 and DN. This may indicate that the locus is of importance in the European populations. Our results further support the previous linkage findings between DN and chromosomes 3q21–q25 and 19q13 and implicate several other possible loci in the pathogenesis of DN in T1D.

## Supporting Information

Table S1 Summary of the non-parametric linkage results for diabetic nephropathy on the regions showing LOD scores 1.00–1.73.
(DOC)

Table S2 List of participating centers and people involved.
(XLS)
