# Supplementary material for: Novel Susceptibility Locus at 22q11 for Diabetic Nephropathy in Type 1 Diabetes
Source: PLoS One. 2011 Sep 1;6(9):e24053. doi: 10.1371/journal.pone.0024053 (PMC3164698; doi:10.1371/journal.pone.0024053)
Supplement: Table S1 — Summary of the non-parametric linkage results for diabetic nephropathy on the regions showing LOD scores 1.00–1.73. (DOC) [file pone.0024053.s001.doc]

Table S1 Summary of the non-parametric linkage results for diabetic nephropathy on the regions showing LOD scores 1.00 – 1.73.

|  |  |  |  | Non-parametric two-point | | Non-parametric multipoint | |
| --- | --- | --- | --- | --- | --- | --- | --- |
|  | Chr | cM | Marker | ASP LOD | All-ASP LOD | NPL_pair_ LOD | NPL_qtl_ LOD |
| Finnish | 3q24 | 150.6 | D3S1569 | 0.76 | 1.08 | 1.07 | 1.36 |
|  | 4q13.3 | 80.38 | D4S392 | 1.00 | 1.36 | 0.86 | 1.37 |
|  | 7q21.11 | 89.93 | D7S669 | 0.01 | 1.73 | 0.01 | 0.81 |
|  | 8q24.23 | 150.14 | D8S272 | 0.53 | 1.27 | 0.95 | 0.9 |
|  | 11p15.1 | 25.69 | D11S902 | 1.46 | 0.59 | 1.40 | 0.32 |
|  | 15q14 | 38.12 | D15S1012 | 0.22 | 1.19 | 0.32 | 1.42 |
|  | 15q21.1 | 48.18 | D15S978 | 0.91 | 0.66 | 1.15 | 1.56 |
|  | 15q22.1 | 54.46 | D15S117 | 1.17 | 0.26 | 1.60 | 0.69 |
|  | 18p11 | 37.67 | D18S53 | 0.19 | 0.00 | 1.22 | 0.06 |
|  | 19q13.43 | 108.6 | D19S210 | 1.61 | 0.12 | 1.51 | 0.33 |
|  | 21q22.3 | 56 | D21S266 | 0.06 | 0.6 | 0.02 | 1.07 |
|  | 22q11.23-q12.1 | 22.59 | D22S315 | 0.00 | 0.77 | 0.0 | 1.29 |
| Danish | 1q43 | 240.77 | D1S2800 | 1.15 | 0.43 | 0.94 | 0.31 |
|  | 3q25 | 160.19 | D3S1279 | 1.13 | 0.89 | 0.54 | 0.55 |
|  | 3q29 | 220.19 | D3S1311 | 0.42 | 1.11 | 0.09 | 0.67 |
|  | 5q33.2 | 158.44 | D5S410 | 0.2 | 0.81 | 0.12 | 1.15 |
|  | 6q14 | 90.58 | D6S460 | 1.39 | 0.06 | 1.20 | 0.02 |
|  | 6q15 | 97.76 | D6S462 | 1.13 | 0.00 | 1.14 | 0.00 |
|  | 6q27 | 189.16 | D6S446 | 0.00 | 1.25 | -0.02 | 0.96 |
|  | 7q11 | 79.12 | D7S502 | 1.15 | 0.06 | 1.48 | 0.07 |
|  | 10p13 | 37.75 | D10S1653 | 0.98 | 0.02 | 1.35 | 0.33 |
|  | 10p12 | 43.4 | D10S548 | 0.55 | 0.03 | 1.21 | 0.28 |
|  | 22q11 | 2.96 | D22S420 | 0.66 | 0.59 | 1.35 | 1.15 |
|  | 22q11.21-q11.22 | 14.68 | D22S539 | 0.72 | 0.19 | 1.30 | 0.57 |
|  | 22q12.3 | 42.07 | D22S283 | 1.11 | 0.00 | 0.57 | -0.19 |
|  | 22q13.31 | 56.47 | D22S274 | 1.14 | 0.00 | 0.84 | -0.01 |
| French | 2q37 | 240.03 | D2S206 | 1.19 | 0.09 | 0.04 | -0.01 |
|  | 6p23 | 34.61 | D6S289 | 1.36 | 2.61 | 1.18 | 1.20 |
|  | 7q36.3 | 181.64 | D7S2465 | 0.99 | 0.19 | 1.16 | 0.21 |
|  | 9p21.3 | 45.57 | D9S171 | 0.09 | 0.43 | 0.61 | 1.46 |
|  | 9p13 | 56.48 | D9S1817 | 0.51 | 0.24 | 1.09 | 1.12 |
|  | 9q21 | 81.01 | D9S167 | 1.36 | 0.90 | 1.42 | 0.91 |
|  | 9q22.32 | 98.7 | D9S287 | 0.05 | 0.05 | 1.05 | 0.38 |
|  | 9q31.1 | 104.08 | D9S1690 | 0.71 | 0.08 | 1.38 | 0.18 |
|  | 9q31.3 | 112.85 | D9S1677 | 1.45 | 0.47 | 1.64 | 0.14 |
|  | 9q33.1 | 121.62 | D9S1776 | 1.68 | 0.02 | 1.5 | 0.08 |
|  | 10q26 | 171.95 | D10S1651 | 1.22 | 0.22 | 1.27 | 0.06 |
|  | 10q26 | 177.19 | D10S212 | 1.20 | 1.04 | 1.37 | 0.39 |
|  | 16q12 | 66.97 | D16S415 | 0.65 | 0.01 | 1.11 | -0.00 |
|  | 17p13 | 30.67 | D17S1852 | 1.58 | 0.35 | 1.31 | 0.18 |
|  | 17p12 | 37 | D17S799 | 0.33 | 0.002 | 1.00 | 0.12 |
|  | 17p12 | 41.47 | D17S921 | 0.00 | 0.00 | 1.09 | 0.24 |
|  | 17p11 | 46.39 | D17S1857 | 1.30 | 0.62 | 1.17 | 0.46 |
|  | 22q11.21-q11.22 | 14.68 | D22S539 | 1.59 | 0.15 | 1.36 | 0.03 |
|  | 22q11.23-q12.1 | 22.59 | D22S315 | 0.75 | 0.00 | 1.28 | 0.00 |
| Joint | 4q13.3 | 80.38 | D4S392 | 0.67 | 0.8 | 1.05 | 1.24 |
|  | 4q21.21 | 88.45 | D4S2964 | 0.25 | 0.69 | 0.42 | 1.44 |
|  | 4q21.23 | 93.19 | D4S1534 | 0.00 | 0.46 | 0.10 | 1.00 |
|  | 4q22.1 | 100.16 | D4S414 | 0.02 | 1.25 | 0.07 | 1.54 |
|  | 5q33.1 | 158.44 | D5S410 | 0.00 | 0.58 | -0.04 | 1.36 |
|  | 5q34 | 166.12 | D5S422 | 0.12 | 0.59 | 0.02 | 1.04 |
|  | 6p21.2 | 58.47 | D6S1610 | 0.87 | 0.00 | 1.36 | -0.02 |
|  | 6p12.1 | 79.52 | D6S257 | 0.55 | 0.00 | 1.03 | -0.07 |
|  | 6q27 | 189.16 | D6S446 | 0.00 | 0.30 | -0.07 | 1.04 |
|  | 7q21.11 | 89.39 | D7S669 | 0.14 | 1.92 | 0.23 | 0.75 |
|  | 7q36.1 | 162.09 | D7S636 | 0.65 | 0.002 | 1.26 | 0.28 |
|  | 9q31.3 | 112.85 | D9S1677 | 1.70 | 0.09 | 0.54 | 0.01 |
|  | 11p14.2 | 43.64 | D11S904 | 1.01 | 0.00 | 0.67 | -0.01 |
|  | 17q25.3 | 129.62 | D17S784 | 0.40 | 1.37 | 0.72 | 0.68 |
|  | 20p13 | 11.93 | D20S889 | 0.00 | 1.00 | -0.03 | 0.26 |

Chr=chromosome; ASP = non-parametric two-point affected sib-pair LOD score; NPL = non-parametric multipoint LOD score based on Merlin sib-pair analysis; NPL_qtl_ = non-parametric multipoint LOD score based on Merlin qtl-analysis
